# The complete chloroplast genome sequence of *Prunus simonii* ‘Weiwang’

**DOI:** 10.1080/23802359.2021.1950060

**Published:** 2021-07-15

**Authors:** Yicen Xu, Bo Fang, Jie Yu

**Affiliations:** a College of Horticulture and Landscape Architecture, Southwest University, Chongqing, China; b Chongqing Academy of Agricultural Sciences, Chongqing, China

**Keywords:** *Prunus simonii* ‘Weiwang’, chloroplast genome, phylogenetic analysis

## Abstract

*Prunus simonii* ‘Weiwang’ is an important economic fruit crops. In this study, we reported the complete chloroplast genome sequence of *P. simonii* ‘Weiwang’. The genome has a circular structure of 157,924 bp containing a large single-copy region (LSC) of 86,187 bp, a small copy region (SSC) of 19,031 bp, and two inverted repeats (IR) of 26,353 bp by each. It harbors 110 unique genes, including 78 protein-coding genes, 4 ribosomal RNA genes, and 28 transfer RNA genes. The phylogenomic analysis shows that *Prunus simonii* ‘Weiwang’ is clustered with *Prunus salicina*.

*Prunus simonii* ‘Weiwang’ is a successful hybrid cultivated by American agronomists over half a century. It belongs to *Prunus* of Rosaceae. The fruit has good characters in shape and color, fragrant, sour and sweet in taste, and nutritious. It comes into the market in summer, with early ripening and high yield, which is favored by consumers and growers (Xianzhong et al. 2011).

*Prunus simonii* ‘Weiwang’ was collected from Sanjiao town, Qijiang District, Chongqing. The DNA library was constructed using the Agilent 2100 and sequenced by using the Illumina NovaSeq 6000 sequencing platform.

The chloroplast genome was assembled from the clean data by GetOrganelle (v. 1.6.4) (Jin et al. 2020). The correctness of the assembly was confirmed by using Bowtie2 (v2. 0.1) (Langmead et al. 2009). The annotation of the chloroplast genome was conducted initially using CpGAVAS2 (Linchun et al. 2019). Furthermore, the annotations with problems were manually edited by using Apollo (Misra and Harris 2006). The genome sequence and annotations have been deposited in the GenBank with accession number MW406463.

The chloroplast genome of *P. simonii* ‘Weiwang’ is 157,924 bp in size with a large single-copy region (LSC) of 86,187 bp, small copy region (SSC) of 19,031 bp and two inverted repeats (IRs) of 26,353 bp by each. The chloroplast genome of *P. simonii* ‘Weiwang’ comprises 131 genes, among which, 110 are unique genes, including 78 protein-coding genes, 4 ribosomal RNA (rRNA) genes, and 28 transfer RNA (tRNA) genes. Among the 78 protein coding genes annotated, nine unique genes (*rps*16, *atp*F, *rpo*C1, *pet*B, *pet*D, *rpl*16, *rpl*2, *ndh*B, *ndh*A) contain only one intron, two genes (*ycf*3, *clp*P) contain two introns, and six tRNA genes (*trn*K-UUU, *trn*G-GCC, *trn*L-UAA, *trn*V-UAC, *trn*I-GAU, *trn*A-UGC) contain one intron. The GC content analysis showed that the overall GC content is 36.72%. Note that the GC contents in IR regions (42.62%) are significantly higher than that in LSC (34.51%) and SSC regions (30.37%).

To examine the phylogenetic position of *P. simonii* ‘Weiwang’, we constructed the maximum likelihood (ML) trees using the complete chloroplast genomes of *P. simonii* ‘Weiwang’ ,other 29 *Prunus* species, and two outgroups. The complete chloroplast genome sequences were aligned by using MAFFT (https://mafft.cbrc.jp/alignment/server/) online version 7.471 (John et al. 2019). These aligned sequences were used to construct the maximum likelihood tree by RaxML (v8.2.4) (Stamatakis 2014). The phylogenetic analysis showed *P. simonii* ‘Weiwang’ was clustered with *Prunus salicina* (Figure 1).

**Figure 1.**
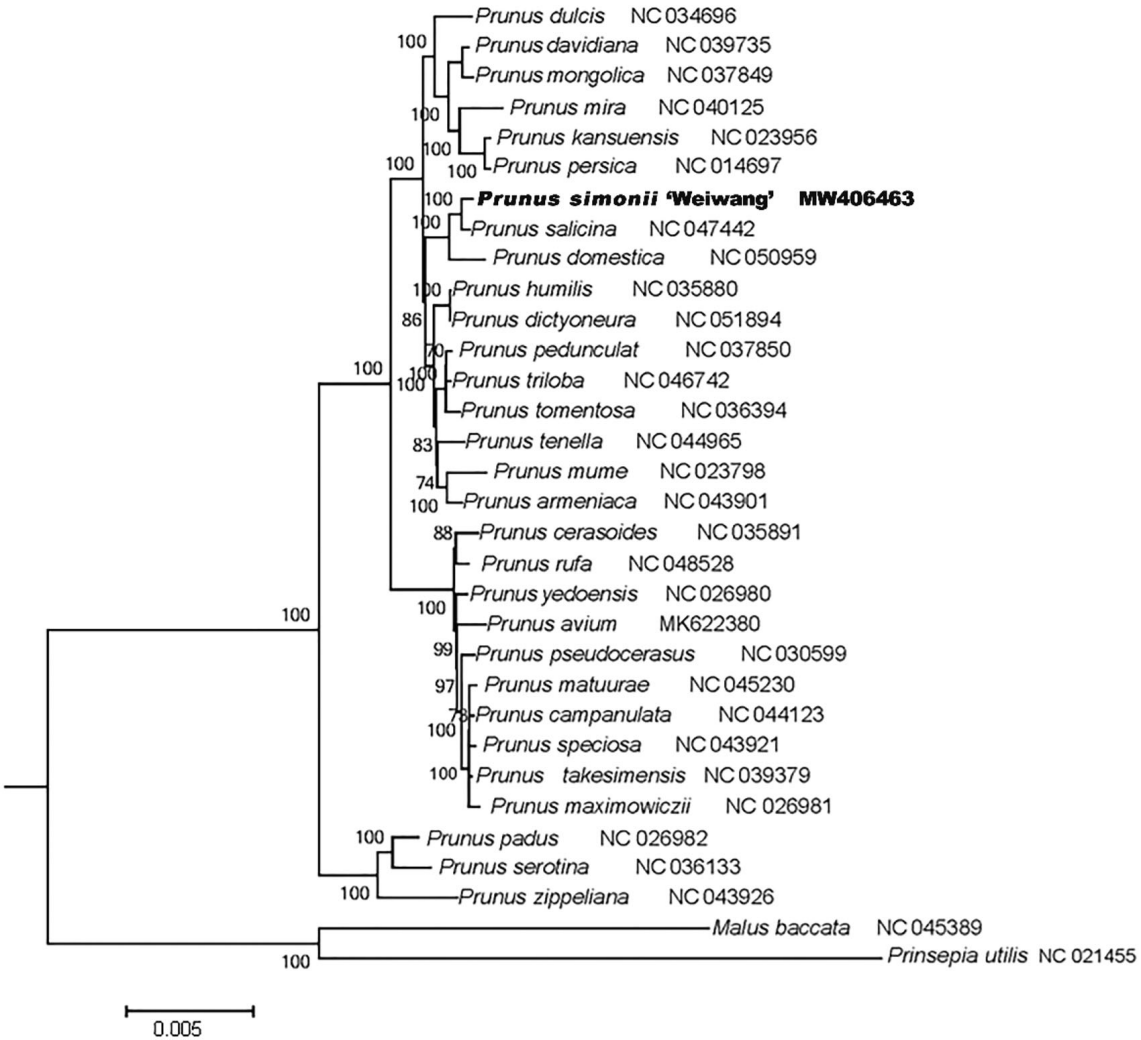
Maximum-likelihood phylogenetic tree for genus *Prunus* L. based on 32 complete chloroplast genomes. Values along branches correspond to ML bootstrap percentages.

## Data Availability

The genome sequence data that support the findings of this study are openly available in GenBank of NCBI at https://www.ncbi.nlm.nih.gov/ under the accession number MW406463. The associated “BioProject,” “SRA,” and “Bio-Sample” numbers are PRJNA719267, SRR14133449, and SAMN18593728 respectively.”
